# (*E*)-2-[1-(4-Fluoro­phen­yl)pent-1-en-3-yl­idene]malononitrile

**DOI:** 10.1107/S1600536811035884

**Published:** 2011-09-14

**Authors:** Tai-Ran Kang

**Affiliations:** a College of Chemistry and Chemical Engineering, China West Normal University, Nanchong 637002, People’s Republic of China

## Abstract

The title mol­ecule, C_14_H_11_FN_2_, is approximately planar except the ethyl group, the maximum atomic deviation being 0.105 (5) Å. The fluoro­phenyl ring and 2-propyl­idene­malononitrile unit are located on the opposite sides of the C=C double bond, showing an *E* configuration.

## Related literature

The title compound is a diene reagent in Diels–Alder reactions. For the use of malononitrile-containing compounds as building blocks in organic synthesis, see: Liu *et al.* (2002 ▶); Sepiol & Milart (1985 ▶); Zhang *et al.* (2003 ▶). For related structures, see: Kang & Chen (2009 ▶).
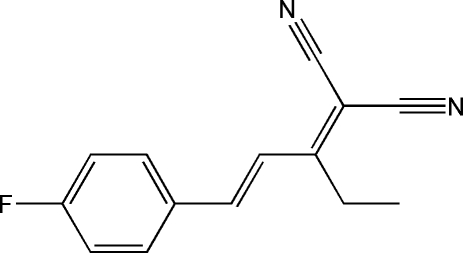


## Experimental

### 

#### Crystal data

C_14_H_11_FN_2_
*M* *_r_* = 226.25
Monoclinic, 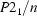

*a* = 7.6504 (2) Å
*b* = 12.4989 (3) Å
*c* = 12.7787 (3) Å
β = 98.375 (2)°
*V* = 1208.89 (5) Å^3^
*Z* = 4
Cu *K*α radiation
μ = 0.70 mm^−1^
*T* = 291 K
0.42 × 0.38 × 0.32 mm

#### Data collection

Oxford Diffraction Xcalibur Sapphire3 Gemini ultra diffractometer
Absorption correction: multi-scan (*CrysAlis PRO*; Oxford Diffraction, 2009 ▶) *T*_min_ = 0.758, *T*_max_ = 0.808
5033 measured reflections
2148 independent reflections
1956 reflections with *I* > 2σ(*I*)
*R*_int_ = 0.014

#### Refinement

*R*[*F* ^2^ > 2σ(*F* ^2^)] = 0.041
*wR*(*F* ^2^) = 0.117
*S* = 1.06
2148 reflections
155 parameters
H-atom parameters constrained
Δρ_max_ = 0.10 e Å^−3^
Δρ_min_ = −0.14 e Å^−3^

### 

Data collection: *CrysAlis PRO* (Oxford Diffraction, 2009 ▶); cell refinement: *CrysAlis PRO*; data reduction: *CrysAlis PRO*; program(s) used to solve structure: *SHELXTL* (Sheldrick, 2008 ▶); program(s) used to refine structure: *SHELXTL*; molecular graphics: *SHELXTL*; software used to prepare material for publication: *SHELXTL*.

## Supplementary Material

Crystal structure: contains datablock(s) global, I. DOI: 10.1107/S1600536811035884/xu5286sup1.cif

Structure factors: contains datablock(s) I. DOI: 10.1107/S1600536811035884/xu5286Isup2.hkl

Supplementary material file. DOI: 10.1107/S1600536811035884/xu5286Isup3.cml

Additional supplementary materials:  crystallographic information; 3D view; checkCIF report

## Figures and Tables

**Fig. 1.**
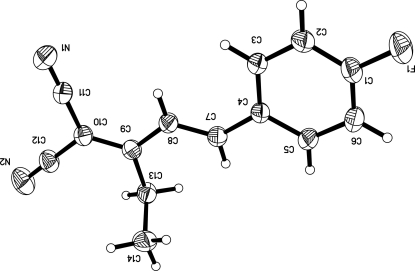
The molecular structure of (I) with 30% probability displacement ellipsoids (arbitrary spheres for H atoms).

## supplementary crystallographic information

### Comment

The chemistry of ylidene malononitrile have been studied extensively, From the ring closure reactions, the comounds containing newly formed five or six-membered rings, such as indans (Zhang *et al.*, 2003), naphthalenes (Liu *et al.*, 2002), benzenes (Sepiol & Milart, 1985) were obtained. Some crystal structures involving ylidene malononitrile groups have been published, including a recent report from our labratory (Kang & Chen, 2009). As a part of our interest in the synthsis of some complex ring systems, we investigated the title compound (I), which is a diene reagent in Diels-Alder reaction. We report herein the crystal structure of the title compound.

The molecular structure of (I) is shown in Fig. 1. Bond lengths and angles in (I) are normal. The phenyl ring with two double bond and triple bond is copolar.The fluorophenyl ring and 2-propylidenemalononitrile groups are located on opposite sides of the double bond, showing an E configuration.

### Experimental

2-(Butan-2-ylidene)malononitrile (0.24 g, 2 mmol) and 4-fluorobenzaldehyde (0.248 g, 2 mmol) were dissolved in 2-propanol (2 ml). To the solution was added piperidine (0.017 g, 0.2 mmol), the solution was stirred for 24 h at 343 K. Then the reaction was cooled to room temperature, and the solution was filtered to obtain a white solid. Recrystallization from hot ethanol afforded the pure compound. Single crystals of (I) suitable for X-ray analysis were obtained by slow evaporation ethanol solvent.

### Refinement

The carbon-bound H atoms were placed in calculated positions, with C—H = 0.93–0.97 Å, and refined using a riding model, with *U*_iso_(H) =1.5*U*_eq_(C) for methyl H atoms and *U*_iso_(H) = 1.2*U*_eq_(C) for the others.

### Crystal data

**Table:**

C_14_H_11_FN_2_	*F*(000) = 472
*M**_r_* = 226.25	*D*_x_ = 1.243 Mg m^−^^3^
Monoclinic, *P*2_1_/*n*	Cu *K*α radiation, λ = 1.54184 Å
Hall symbol: -P 2yn	Cell parameters from 3458 reflections
*a* = 7.6504 (2) Å	θ = 3.5–71.8°
*b* = 12.4989 (3) Å	µ = 0.70 mm^−^^1^
*c* = 12.7787 (3) Å	*T* = 291 K
β = 98.375 (2)°	Block, yellow
*V* = 1208.89 (5) Å^3^	0.42 × 0.38 × 0.32 mm
*Z* = 4	

### Data collection

**Table:**

Oxford Diffraction Xcalibur Sapphire3 Gemini ultra diffractometer	2148 independent reflections
Radiation source: Enhance Ultra (Cu) X-ray Source	1956 reflections with *I* > 2σ(*I*)
mirror	*R*_int_ = 0.014
Detector resolution: 15.9149 pixels mm^-1^	θ_max_ = 67.1°, θ_min_ = 5.0°
ω scans	*h* = −9→8
Absorption correction: multi-scan (*CrysAlis PRO*; Oxford Diffraction, 2009)	*k* = −14→11
*T*_min_ = 0.758, *T*_max_ = 0.808	*l* = −15→13
5033 measured reflections	

### Refinement

**Table:**

Refinement on *F*^2^	Primary atom site location: structure-invariant direct methods
Least-squares matrix: full	Secondary atom site location: difference Fourier map
*R*[*F*^2^ > 2σ(*F*^2^)] = 0.041	Hydrogen site location: inferred from neighbouring sites
*wR*(*F*^2^) = 0.117	H-atom parameters constrained
*S* = 1.06	*w* = 1/[σ^2^(*F*_o_^2^) + (0.0672*P*)^2^ + 0.0976*P*] where *P* = (*F*_o_^2^ + 2*F*_c_^2^)/3
2148 reflections	(Δ/σ)_max_ = 0.005
155 parameters	Δρ_max_ = 0.10 e Å^−^^3^
0 restraints	Δρ_min_ = −0.14 e Å^−^^3^

### Special details

**Table:**

Geometry. All e.s.d.'s (except the e.s.d. in the dihedral angle between two l.s. planes) are estimated using the full covariance matrix. The cell e.s.d.'s are taken into account individually in the estimation of e.s.d.'s in distances, angles and torsion angles correlations between e.s.d.'s in cell parameters are only used when they are defined by crystal symmetry. An approximate (isotropic) treatment of cell e.s.d.'s is used for estimating e.s.d.'s involving l.s. planes.
Refinement. Refinement of *F*^2^ against ALL reflections. The weighted *R*-factor *wR* and goodness of fit *S* are based on *F*^2^, conventional *R*-factors *R* are based on *F*, with *F* set to zero for negative *F*^2^. The threshold expression of *F*^2^ > σ(*F*^2^) is used only for calculating *R*-factors(gt) *etc*. and is not relevant to the choice of reflections for refinement. *R*-factors based on *F*^2^ are statistically about twice as large as those based on *F*, and *R*- factors based on ALL data will be even larger.

### Fractional atomic coordinates and isotropic or equivalent isotropic displacement parameters (Å^2^)

**Table:**

	*x*	*y*	*z*	*U*_iso_*/*U*_eq_	
C11	0.29662 (18)	−0.17966 (10)	0.91075 (11)	0.0583 (3)	
F1	0.17654 (14)	0.52071 (7)	0.95680 (10)	0.0992 (4)	
C9	0.49407 (15)	−0.06063 (10)	0.82906 (9)	0.0500 (3)	
C4	0.36822 (15)	0.23270 (9)	0.86946 (9)	0.0483 (3)	
N1	0.18077 (18)	−0.19553 (12)	0.95555 (12)	0.0819 (4)	
C5	0.45722 (17)	0.32820 (10)	0.85718 (11)	0.0595 (3)	
H5	0.5624	0.3264	0.8288	0.071*	
C13	0.65462 (17)	−0.04859 (10)	0.77465 (10)	0.0571 (3)	
H13A	0.6384	0.0122	0.7270	0.069*	
H13B	0.6677	−0.1120	0.7327	0.069*	
C3	0.21017 (16)	0.23790 (10)	0.91178 (9)	0.0515 (3)	
H3	0.1477	0.1754	0.9201	0.062*	
C14	0.82131 (18)	−0.03252 (13)	0.85331 (12)	0.0704 (4)	
H14A	0.8075	0.0290	0.8964	0.106*	
H14B	0.9199	−0.0217	0.8158	0.106*	
H14C	0.8420	−0.0947	0.8975	0.106*	
C12	0.53656 (19)	−0.25427 (10)	0.83029 (11)	0.0611 (4)	
C7	0.44770 (16)	0.13350 (9)	0.83921 (10)	0.0520 (3)	
H7	0.5433	0.1410	0.8024	0.062*	
C10	0.44336 (16)	−0.16038 (9)	0.85528 (10)	0.0523 (3)	
C2	0.14552 (18)	0.33429 (10)	0.94140 (11)	0.0593 (3)	
H2	0.0406	0.3374	0.9700	0.071*	
C1	0.23896 (19)	0.42570 (10)	0.92788 (12)	0.0633 (4)	
C8	0.40019 (15)	0.03307 (9)	0.85783 (9)	0.0509 (3)	
H8	0.3012	0.0228	0.8912	0.061*	
C6	0.39336 (19)	0.42521 (10)	0.88608 (12)	0.0658 (4)	
H6	0.4536	0.4885	0.8774	0.079*	
N2	0.6121 (2)	−0.32851 (10)	0.81089 (12)	0.0867 (5)	

### Atomic displacement parameters (Å^2^)

**Table:**

	*U*^11^	*U*^22^	*U*^33^	*U*^12^	*U*^13^	*U*^23^
C11	0.0574 (7)	0.0436 (6)	0.0741 (8)	−0.0017 (5)	0.0100 (6)	0.0036 (5)
F1	0.1059 (7)	0.0510 (5)	0.1490 (10)	0.0130 (5)	0.0465 (7)	−0.0189 (5)
C9	0.0499 (6)	0.0461 (6)	0.0545 (6)	0.0027 (5)	0.0096 (5)	−0.0024 (5)
C4	0.0484 (6)	0.0419 (6)	0.0560 (6)	0.0011 (5)	0.0119 (5)	0.0056 (5)
N1	0.0709 (8)	0.0697 (8)	0.1101 (10)	−0.0068 (6)	0.0300 (7)	0.0134 (7)
C5	0.0540 (7)	0.0479 (7)	0.0808 (9)	−0.0026 (5)	0.0244 (6)	0.0060 (6)
C13	0.0607 (7)	0.0498 (7)	0.0650 (7)	0.0045 (5)	0.0232 (6)	−0.0012 (5)
C3	0.0504 (6)	0.0452 (6)	0.0602 (7)	−0.0003 (5)	0.0131 (5)	0.0067 (5)
C14	0.0541 (7)	0.0799 (10)	0.0803 (9)	0.0043 (6)	0.0203 (6)	0.0063 (7)
C12	0.0687 (8)	0.0465 (7)	0.0672 (8)	0.0053 (6)	0.0064 (6)	−0.0044 (6)
C7	0.0501 (6)	0.0472 (7)	0.0617 (7)	0.0028 (5)	0.0179 (5)	0.0031 (5)
C10	0.0539 (7)	0.0426 (6)	0.0606 (7)	0.0020 (5)	0.0091 (5)	−0.0019 (5)
C2	0.0557 (7)	0.0562 (8)	0.0694 (8)	0.0066 (5)	0.0203 (6)	0.0020 (6)
C1	0.0691 (8)	0.0453 (7)	0.0774 (8)	0.0094 (6)	0.0166 (6)	−0.0048 (6)
C8	0.0503 (6)	0.0436 (6)	0.0615 (7)	0.0018 (5)	0.0170 (5)	0.0013 (5)
C6	0.0701 (8)	0.0417 (7)	0.0878 (9)	−0.0069 (6)	0.0190 (7)	0.0015 (6)
N2	0.1048 (11)	0.0568 (8)	0.0969 (10)	0.0233 (7)	0.0096 (8)	−0.0131 (6)

### Geometric parameters (Å, °)

**Table:**

C11—N1	1.1404 (18)	C3—C2	1.3762 (17)
C11—C10	1.4326 (19)	C3—H3	0.9300
F1—C1	1.3514 (15)	C14—H14A	0.9600
C9—C10	1.3620 (17)	C14—H14B	0.9600
C9—C8	1.4489 (16)	C14—H14C	0.9600
C9—C13	1.5045 (17)	C12—N2	1.1393 (18)
C4—C5	1.3943 (16)	C12—C10	1.4331 (17)
C4—C3	1.3961 (16)	C7—C8	1.3378 (16)
C4—C7	1.4577 (16)	C7—H7	0.9300
C5—C6	1.3777 (18)	C2—C1	1.372 (2)
C5—H5	0.9300	C2—H2	0.9300
C13—C14	1.518 (2)	C1—C6	1.365 (2)
C13—H13A	0.9700	C8—H8	0.9300
C13—H13B	0.9700	C6—H6	0.9300
			
N1—C11—C10	179.39 (16)	C13—C14—H14C	109.5
C10—C9—C8	120.53 (11)	H14A—C14—H14C	109.5
C10—C9—C13	119.09 (11)	H14B—C14—H14C	109.5
C8—C9—C13	120.31 (10)	N2—C12—C10	179.37 (16)
C5—C4—C3	117.93 (11)	C8—C7—C4	128.05 (11)
C5—C4—C7	117.97 (11)	C8—C7—H7	116.0
C3—C4—C7	124.09 (10)	C4—C7—H7	116.0
C6—C5—C4	121.68 (12)	C9—C10—C11	123.19 (11)
C6—C5—H5	119.2	C9—C10—C12	121.73 (12)
C4—C5—H5	119.2	C11—C10—C12	115.07 (11)
C9—C13—C14	111.77 (11)	C1—C2—C3	118.66 (12)
C9—C13—H13A	109.3	C1—C2—H2	120.7
C14—C13—H13A	109.3	C3—C2—H2	120.7
C9—C13—H13B	109.3	F1—C1—C6	118.11 (12)
C14—C13—H13B	109.3	F1—C1—C2	119.08 (12)
H13A—C13—H13B	107.9	C6—C1—C2	122.81 (11)
C2—C3—C4	120.90 (11)	C7—C8—C9	123.76 (11)
C2—C3—H3	119.5	C7—C8—H8	118.1
C4—C3—H3	119.5	C9—C8—H8	118.1
C13—C14—H14A	109.5	C1—C6—C5	118.00 (12)
C13—C14—H14B	109.5	C1—C6—H6	121.0
H14A—C14—H14B	109.5	C5—C6—H6	121.0
			
C3—C4—C5—C6	0.4 (2)	C13—C9—C10—C12	−1.42 (19)
C7—C4—C5—C6	−178.49 (13)	C4—C3—C2—C1	0.4 (2)
C10—C9—C13—C14	−91.40 (14)	C3—C2—C1—F1	−179.96 (13)
C8—C9—C13—C14	85.53 (14)	C3—C2—C1—C6	0.1 (2)
C5—C4—C3—C2	−0.66 (19)	C4—C7—C8—C9	−176.65 (12)
C7—C4—C3—C2	178.11 (12)	C10—C9—C8—C7	176.67 (12)
C5—C4—C7—C8	169.58 (13)	C13—C9—C8—C7	−0.22 (19)
C3—C4—C7—C8	−9.2 (2)	F1—C1—C6—C5	179.66 (14)
C8—C9—C10—C11	0.61 (19)	C2—C1—C6—C5	−0.4 (2)
C13—C9—C10—C11	177.53 (12)	C4—C5—C6—C1	0.2 (2)
C8—C9—C10—C12	−178.34 (11)		
